# Effect of rapid cefpodoxime disk screening for early detection of third-generation cephalosporin resistance in *Escherichia coli* and *Klebsiella pneumoniae* bacteremia

**DOI:** 10.1186/s40780-023-00313-x

**Published:** 2023-12-01

**Authors:** Mikiyasu Sakai, Takamasa Sakai, Yuki Nagata, Hiroko Komai, Yoshio Miyake, Toshitaka Watariguchi, Atsushi Kawabata, Fumiko Ohtsu

**Affiliations:** 1 Graduate School of Pharmacy, Meijo University, 150 Yagotoyama, Tempaku-Ku, Nagoya, Aichi 468-8503 Japan; 2 Department of Pharmacy, Toyota Kosei Hospital, 500-1, Ibobara, Toyota, Jousui-Cho 470-0396 Japan; 3 Drug Informatics, Faculty of Pharmacy, Meijo University, 150 Yagotoyama, Tempaku-Ku, Nagoya, Aichi 468-8503 Japan; 4 Department of Clinical Laboratory, Toyota Kosei Hospital, 500-1, Ibobara, Toyota, Jousui-Cho 470-0396 Japan; 5 Department of Infection Control, Toyota Kosei Hospital, 500-1, Ibobara, Toyota, Jousui-Cho 470-0396 Japan; 6 Department of General Internal Medicine, Toyota Kosei Hospital, 500-1, Ibobara, Toyota, Jousui-Cho 470-0396 Japan; 7 Department of Infectious Disease, Toyota Kosei Hospital, 500-1, Ibobara, Toyota, Jousui-Cho 470-0396 Japan

**Keywords:** Antimicrobial susceptibility testing, Bacteremia, Antimicrobial resistance

## Abstract

**Background:**

Several methods have been reported for detecting resistance genes or phenotypic testing on the day of positive blood culture in *Escherichia coli* or *Klebsiella pneumoniae* bacteremia. However, some facilities have not introduced these methods because of costs or other reasons. Toyota Kosei Hospital introduced cefpodoxime (CPDX) rapid screening on May 7, 2018, to enable early detection of third-generation cephalosporin resistance. In this study, we aimed to evaluate the effects of intervention with an Antimicrobial Stewardship Team using CPDX rapid screening.

**Methods:**

Cefotaxime (CTX)-resistant *E. coli* or *K. pneumoniae* bacteremia cases were selected retrospectively and divided into two groups: the pre-CPDX screening (June 1, 2015, to May 6, 2018) and CPDX screening groups (July 7, 2018, to August 31, 2021). The primary outcome was the proportion of cases in which modifications were made to the administration of susceptible antimicrobial agents within 24 h of blood culture-positive reports.

**Results:**

Overall, 63 patients in the pre-CPDX screening group and 84 patients in the CPDX screening group were eligible for analysis. The proportion of patients who modified to susceptible antimicrobial agents within 24 h of blood culture-positive reports was significantly increased in the CPDX screening group compared to that in the pre-CPDX screening group (6.3% vs. 22.6%, *p* = 0.010).

**Conclusion:**

The results demonstrated that in CTX-resistant *E. coli* or *K. pneumoniae* bacteremia, CPDX rapid screening increased the proportion of early initiation of appropriate antimicrobial agents.

## Background

In *Escherichia coli* and *Klebsiella pneumoniae*, most third-generation cephalosporin-resistant strains are extended-spectrum β-lactamase (ESBL)-producing bacteria [1], and the number of ESBL-producing bacteria is increasing [2]. Infections caused by third-generation cephalosporin-resistant *E. coli* and *K. pneumoniae*, including ESBL-producing bacteria, are associated with higher mortality [3, 4], higher hospitalization costs, and longer hospital stays than infections caused by non-resistant bacteria [4–7]. Inappropriate empiric therapy is associated with higher mortality in bacteremia caused by ESBL-producing bacteria [8], and appropriate antimicrobial therapy should be initiated earlier. In antimicrobial susceptibility testing of Enterobacterales, it is usually necessary to culture the samples for 16–20 h [9], and patients with bacteremia require approximately one day from a positive blood culture to the determination of antimicrobial resistance. Methods for determining antimicrobial resistance on the day of positive blood culture include rapid genotypic and phenotypic susceptibility testing. Rapid genotypic susceptibility testing includes methods such as the Verigene system [10]; however, it is expensive, with testing costs reported to be $99 per test [11]. Rapid phenotypic susceptibility testing includes methods such as the Accelerate Pheno System [12] and Rapid Antimicrobial Susceptibility Testing (RAST) published by the European Committee on Antimicrobial Susceptibility Testing [13]. Introducing equipment to an Accelerate Pheno System can be expensive, whereas the RAST method can be difficult to introduce because it requires matrix-assisted laser desorption ionization time-of-flight mass spectrometry (MALDI-TOF MS) to identify bacterial species.

Several studies and randomized controlled trials (RCTs) have evaluated the clinical benefits of rapid genotypic and phenotypic susceptibility testing [14–17]. These studies have reported a reduction in time‐to‐appropriate antibiotic therapy compared to conventional antimicrobial susceptibility testing, contributing to the appropriate use of antimicrobial agents. However, many facilities have not introduced these tests due to costs and other reasons.

Owing to the low cost and rapid detection of third-generation cephalosporin-resistant bacteria, Toyota Kosei Hospital introduced cefpodoxime (CPDX) rapid screening on May 7, 2018. This method is a rapid phenotypic susceptibility test using antimicrobial disks, and it has been reported that the disk diffusion method can read the inhibition zone in a 6 h incubation time [18, 19], a method developed at our hospital. CPDX is the most sensitive individual indicator cephalosporin for detection of ESBL production [20], therefore. It was used as an antimicrobial disk. In this study, we evaluated the accuracy of CPDX rapid screening, an unconventional method, and investigated the clinical impact of intervention with an AST using CPDX rapid screening.

## Methods

### CPDX rapid screening method

CPDX rapid screening was performed by dispensing each drop of positive blood cultures on a Nissui Separated Plate CA Sheep Blood Agar/Chocolate Agar EX II with vancomycin, and a CPDX disk (10 µg) was placed on Chocolate Agar EX II with vancomycin after streaking with an inoculating loop. Susceptibility was determined from the zone diameter of growth inhibition after at least 6 h of incubation at 35 °C using the criteria for zone diameter in Enterobacterales, as indicated by the Clinical and Laboratory Standards Institute (CLSI).

### Blood culture testing and result reporting procedure

All blood culture test were performed by the microbiology laboratory. However, if blood culture positive after microbiology laboratory operating hours, the clinical laboratory technician on duty performed subculture and placed CPDX disk. All blood culture positive report was performed by the microbiology laboratory. The results were entered into the medical record and reported to the primary care physician, AST physician and AST pharmacist. CPDX screening tests and reports were performed according to Table 1. The microbiology laboratory operated from 8:30 a.m. to 5:00 p.m. on weekdays and Saturdays and from 8:30 a.m. to 12:20 p.m. on Sundays and holidays.

**Table 1:** CPDX rapid screening testing and result-reporting protocol

Cases	Testing and result reporting
**Blood culture positive during microbiology laboratory operating hours**	
Weekdays and Saturdays until 11:00 a.m	Perform CPDX screening and report when it is available for determination
Weekdays and Saturdays after 11:00 a.m	Do not perform CPDX screening
Sundays and Holidays	Do not perform CPDX screening
**Blood culture positive after microbiology laboratory operating hours**	
Performed subculture at least 6 h before microbiology laboratory operating hours	Report blood culture and CPDX screening results during the operating hours of the microbiology laboratory
Performed subculture within 6 h before microbiology laboratory operating hours	Report CPDX screening results when it is available for determination during the operating hours of the microbiology laboratory

### Antimicrobial stewardship

In our hospital, Antimicrobial Stewardship Team (AST) physicians or pharmacists reviewed medical records of blood culture-positive patients and suggested antimicrobial modification when necessary, on weekdays. AST physicians or pharmacist suggested antimicrobial modification according to Table 2 until the blood culture was positive and before the antimicrobial susceptibility testing result were available. After the introduction of CPDX rapid screening, AST physicians or pharmacist suggested antimicrobial modification according to Table 3 when CPDX rapid screening identified antimicrobial resistance.

**Table 2:** Antimicrobial prescription protocol for suspected bacteremia caused by Enterobacterales

Cases	Recommendations
**Antimicrobials administered**	
Carbapenems, TAZ/PIPC, TAZ/CTLZ	Continuation of antimicrobials
Aminoglycoside	Continuation of antimicrobials If not urinary tract infection, change to 3rd generation cephalosporins If deemed necessary by the anaerobic activity, change to cephamycins, oxacefems, or TAZ/PIPC However, in severe cases such as septic shock, change to carbapenems
SBT/ABPC, Cephamycins, Oxacephems	Continuation of antimicrobials. ^a^ However, in severe cases such as septic shock, change to carbapenems
Cephalosporins (excluding Cephamycins and Oxacefems), quinolones	Continuation of antimicrobials. ^a^ If deemed necessary by the anaerobic activity, combine with MNZ or change to cephamycins, oxacefems, or TAZ/PIPC However, in severe cases such as septic shock, change to carbapenems
Other antimicrobial agents	Change to 3rd generation cephalosporins If deemed necessary by the anaerobic activity, change to 3rd generation cephalosporins plus MNZ, cephamycins, oxacefems, or TAZ/PIPC However, in severe cases such as septic shock, change to carbapenems
**No antimicrobials administered**	Initiate 3rd generation cephalosporins If deemed necessary by the anaerobic activity, initiate cephamycins, oxacefems, or TAZ/PIPC However, in severe cases such as septic shock, initiate carbapenems

*TAZ/PIPC* Tazobactam/piperacillin, *TAZ/CTLZ* Tazobactam/ceftolozane, *SBT/ABPC* Sulbactam/ampicillin, *MNZ* Metronidazole

^a^If the bacterial species are identified by matrix-assisted laser desorption ionization time-of-flight mass spectrometry and intrinsic resistance to the administered antimicrobial agent is evident, change to an effective antimicrobial agent is suggested

**Table 3:** Antimicrobial prescription protocol when 3rd generation cephalosporin resistance is determined by CPDX screening results

Cases	Recommendations
**Antimicrobials administered**	
Carbapenems	Continuation of antimicrobials
TAZ/PIPC, TAZ/CTLZ, Cephamycins, Oxacephems	Continuation of antimicrobials. ^a^ However, in severe cases such as septic shock, change to carbapenems
Aminoglycoside	Continuation of antimicrobials However, in severe cases such as septic shock and in cases other than urinary tract infection, change to carbapenems
Other antimicrobial agents	Change to carbapenems
**No antimicrobials administered**	Initiate carbapenems

*TAZ/PIPC* Tazobactam/piperacillin, *TAZ/CTLZ* Tazobactam/ceftolozane

^a^If the bacterial species are identified by matrix-assisted laser desorption ionization time-of-flight mass spectrometry and intrinsic resistance to the administered antimicrobial agent is evident, change to an effective antimicrobial agent is suggested

### Patients

Patients with cefotaxime (CTX)-resistant *E. coli* or *K. pneumoniae* detected in blood cultures collected between June 1, 2015, and August 31, 2021, at Toyota Kosei Hospital (a 606-bed tertiary care hospital) were included. Cases of CTX-resistant *E. coli* or *K. pneumoniae* bacteremia that occurred more than once during the study period were included in the primary case only. The exclusion criteria were patients 1) under 18 years of age, 2) with polymicrobial bacteremia, 3) who died within 48 h of blood culture collection, 4) who were transferred during treatment, 5) who were not treated due to palliative care, 6) who were not treated due to diagnosed contamination, and 7) who were not hospitalized. Since CPDX rapid screening was introduced on May 7, 2018, the pre-CPDX screening group included patients between June 1, 2015, to May 6, 2018, and the CPDX screening group included patients between July 7, 2018, to August 31, 2021. The period from May 7, 2018, to July 6, 2018, was excluded because the CPDX screening techniques and reporting system were not considered proficient.

### Evaluation of the accuracy of CPDX rapid screening method

To evaluate the accuracy of CPDX rapid screening method, samples in which *E. coli* and *K. pneumoniae* were detected in the blood cultures collected from July 7, 2018 to August 31, 2021, and for which CPDX rapid screening was performed, the CPDX rapid screening and CTX susceptibility test results were compared. Result comparisons were evaluated by categorical error, with very major error (VME; CPDX rapid screening = S and CTX = R), major error (ME; CPDX rapid screening = R and CTX = S), and minor error (mE; CPDX rapid screening = S or R and CTX = I).

### Data collection

The following data were collected retrospectively from the medical records: age, sex, comorbidity, severity of bacteremia, source of bacteremia, antimicrobial agents, microbiological blood culture results, and susceptibility. Comorbidity and bacteremia severity were evaluated using the Charlson comorbidity index (CCI) and Pitt bacteremia score (PBS), respectively. CCI and PBS were calculated using the data from the positive blood culture collection date. Bacteremia was classified as hospital-acquired if a positive blood culture was obtained from patients who had been hospitalized for 48 h or longer. In cases where CPDX rapid screening was performed, resistance mechanisms (ESBL or AmpC) were also collected. Bacterial species were identified using the MicroScan WalkAway 96 Plus (Beckman Coulter, Japan) until November 11, 2019, and thereafter, using a MALDI Biotyper (Bruker Daltonics, Germany). Antimicrobial susceptibility testing was performed using the MicroScan WalkAway 96 Plus system (Beckman Coulter, Japan).

### Outcomes

**Fig. 1:**
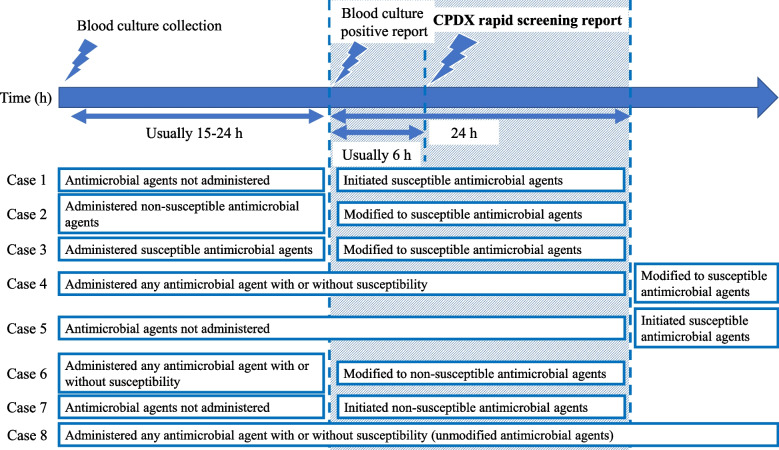
Method of determination of primary outcome. Cases 1–3 were considered as the primary outcomes, while cases 4–7 were not

The primary outcome was the number of cases wherein modifications were made to the administration of susceptible antimicrobial agents within 24 h of blood culture-positive reports. The primary outcome was determined as shown in Fig. 1, with cases 1 to 3 were considered and cases 4 to 8 were not considered. Positive blood culture reports were made by the microbiology laboratory, and information was entered into the medical record at the time of the report. The time for reporting a positive blood culture was defined as the time when information was entered into the medical record. For the time of antimicrobial modification or initiation of antimicrobial agents, the time of initiating antimicrobial agents or after modification was extracted from the medical record. Cases with a difference of 24 h or less between the time of positive blood culture report and the time of antimicrobial modification or initiation were extracted. The secondary outcomes were 30-day mortality, hospital mortality, length of hospital stays from the first positive blood culture collection, and cost of intravenous antibiotic therapy during the bacteremia treatment period. The cost of intravenous antibiotic therapy was calculated by extracting intravenous antimicrobial implementation data from medical records and including antimicrobial solutions using drug prices in Japan as of April 2022.

### Statistical analysis

All statistical analyses were performed using EZR (Saitama Medical Center, Jichi Medical University, Japan) [21]. Continuous variables were tested for normality using the Shapiro–Wilk test. Variables that followed a normal distribution were analyzed using the t-test, and those that did not follow a normal distribution were analyzed using the Mann–Whitney U test. Categorical variables were analyzed using Fisher’s exact test. Statistical significance was set at *p* < 0.05.

## Results

To evaluate the accuracy of the CPDX rapid screening method, 559 samples were included in this study: 425 for *E. coli* and 134 for *K. pneumoniae*. The proportion of categorical agreement was 551 (98.6%), with one (0.18%) for VME, seven (1.25%) for ME, and zero for mE.

**Fig. 2:**
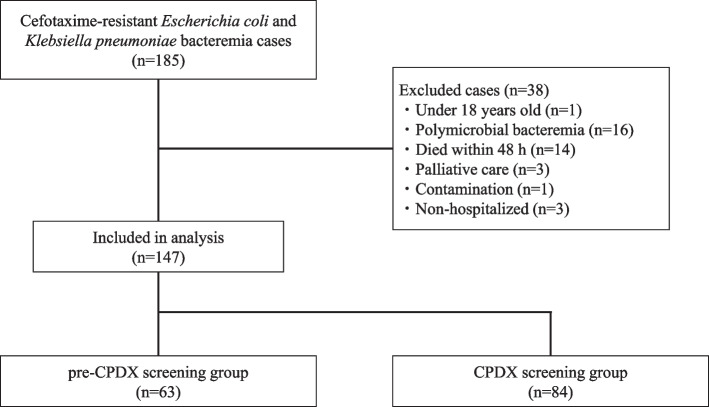
Flow-diagram of patients included in the study. CPDX, cefpodoxime

Of the total 185 cases of CTX-resistant *E. coli* and *K. pneumoniae* bacteremia, 147 were eligible for inclusion: 63 in the pre-CPDX screening group and 84 in the CPDX screening group (Fig. 2). Patient characteristics are shown in Table 4. There was no significant difference between the two groups in each category, and there was no significant difference in the proportion of patients who received a susceptible initial antimicrobial agent before the blood culture positive report (44.4% vs. 46.4%, *p* = 0.868). In the CPDX screening group, the results of CPDX rapid screening were reported in 57 cases (67.9%), and 27 cases (32.1%) did not performed CPDX rapid screening due to microbiology laboratory operating hours. Details of the cases for which CPDX rapid screening was performed are shown in Table 5; 56 (98.2%) were ESBL-producing bacteria and one (1.8%) was AmpC-producing bacteria.

**Table 4:** Characteristics of the patients

	**pre-CPDX screening (*n* = 63)**	**CPDX screening (*n* = 84)**	***p*-value**
**Age (year), median [IQR]**	77 [67.00–85.50]	78 [71.00–85.00]	0.793
**Sex Male, n (%)**	28 (44.4)	45 (53.6)	0.319
**Microorganism, n (%)**			0.214
***Escherichia coli***	52 (82.5)	76 (90.5)	
***Klebsiella pneumoniae***	11 (17.5)	8 (9.5)	
**Hospital-acquired, n (%)**	24 (38.1)	26 (31.0)	0.384
**Pitt bacteremia score, median [IQR]**	1.00 [1.00–3.00]	1.00 [0.00–3.00]	0.903
**≥ 2, n (%)**	27 (42.9)	43 (51.2)	0.404
**Admitted to ICU, n (%)**	1 (1.6)	3 (3.6)	0.635
**Surgical service, n (%)**	1 (1.6)	5 (6.0)	0.238
**Time to positive blood culture report from blood culture collection (h), median [IQR]**	18.29 [15.66–21.30]	18.55 [14.93–22.58]	0.798
**Susceptible initial antimicrobial agent before blood culture positive report, n (%)**	28 (44.4)	39 (46.4)	0.868
**Duration of intravenous antibiotic therapy (days), median [IQR]**	10.00 [7.00–14.00]	8.50 [6.00–13.00]	0.380
**Charlson comorbidity index, median [IQR]**	2.00 [1.00–4.00]	2.00 [1.00–4.00]	0.365
**Source of bacteremia, n (%)**			
**Urinary tract**	39 (61.9)	55 (65.5)	
**Intra-abdominal**	12 (19.0)	18 (21.4)	
**Respiratory**	3 (4.8)	1 (1.2)	
**Others**	1 (1.6)	2 (2.4)	
**Unknown**	8 (12.7)	8 (9.5)	

*IQR* Interquartile range

**Table 5:** Details of the cases in which CPDX rapid screening was performed (*n* = 57)

	**n (%)**
**Microorganism**	
***Escherichia coli***	52 (91.2)
***Klebsiella pneumoniae***	5 (8.8)
**CPDX screening result**	
**Susceptible**	1 (1.8)
**Resistant**	56 (98.2)
**Resistance mechanism**	
**ESBL**	56 (98.2)
**AmpC**	1 (1.8)
**Cefazolin susceptibility**	0 (0)
**Cefmetazole susceptibility**	55 (96.5)

*ESBL* Extended-spectrum β-lactamase

The primary and secondary outcomes are presented in Table 6. For the primary outcome in the CPDX screening group, there was a significant increase in the modified to susceptible antimicrobial agent within 24 h of blood culture positive report (6.3% vs. 22.6%, *p* = 0.01). The details of the primary outcome are shown in Table 7; carbapenems were the main antimicrobial of choice after modification. There were no significant differences in the secondary outcomes of 30-day mortality, hospital mortality, length of hospital stays from blood culture collection, or cost of intravenous antibiotic therapy between the two groups.

**Table 6:** Clinical outcome for patients with cefotaxime-resistant *Escherichia coli* and *Klebsiella pneumoniae* bacteremia cases

	**pre-CPDX screening (n = 63)**	**CPDX screening (n = 84)**	***p*-value**
**Primary outcome**			
**Modifications were made to the administration of susceptible antimicrobial agent within 24 h of blood culture-positive report, n (%)**	4 (6.3)	19 (22.6)	0.010
**Secondary outcome**			
**30-day mortality, n (%)**	4 (6.3)	4 (4.9)	0.728
**Hospital mortality, n (%)**	5 (7.9)	5 (6.1)	0.747
**Length of hospital stays from blood culture collection (days), median [IQR]**	14.00 [11.00–28.50]	13.50 [9.25–26.75]	0.561
**Cost of intravenous antibiotic therapy (yen), median [IQR]**	19035.00 [8410.50–30301.50]	16465.00 [10422.00–24408.00]	0.560

*IQR* Interquartile range

**Table 7:** Details on the primary outcome

	**pre-CPDX screening (*n* = 4)**	**CPDX screening (*n* = 19)**
**Modified, n (%)**		
**SBT/ABPC—> MEPM**	0 (0)	1 (5.3)
**PIPC—> MEPM**	0 (0)	1 (5.3)
**TAZ/PIPC—> MEPM**	0 (0)	2 (10.5)
**CTRX—> MEPM**	1 (25)	7 (36.8)
**SBT/CPZ—> MEPM**	0 (0)	1 (5.3)
**CFPM—> MEPM**	0 (0)	2 (10.5)
**SBT/CPZ—> TAZ/PIPC**	0 (0)	1 (5.3)
**CTRX—> CMZ**	0 (0)	2 (10.5)
**Initiated, n (%)**		
**MEPM**	2 (50)	0 (0)
**CMZ**	0 (0)	2 (10.5)
**SBT/CPZ**	1 (25)	0 (0)

*SBT/ABPC* Sulbactam/ampicillin, *MEPM* Meropenem, *PIPC* Piperacillin, *TAZ/PIPC* Tazobactam/piperacillin, *CTRX* Ceftriaxone, *SBT/CPZ* Sulbactam/cefoperazone, *CFPM* cefepime, *CMZ* Cefmetazole

## Discussion

The CPDX rapid screening accuracy was confirmed by the proportion of categorical agreement for CPDX rapid screening accuracy using VME, ME, and mE which was similar to the RAST accuracy [13]. Therefore, this implies that CPDX rapid screening is a useful method for the early detection of third-generation cephalosporin-resistant bacteria in *E. coli* and *K. pneumoniae* bacteremia. When detecting resistance genes that result in third-generation cephalosporin resistance in *E. coli* and *K. pneumoniae*, AmpC cannot be detected using Verigene or other commonly used genotypic testing. In this study, third-generation cephalosporin resistance due to AmpC production was detected, in only one case using CPDX rapid screening. Therefore, in *E. coli* and *K. pneumoniae* bacteremia, CPDX rapid screening may be more sensitive for third-generation cephalosporin resistance. At the time of the introduction of this method, standardized methods for antimicrobial susceptibility testing directly from blood culture, such as RAST published by EUCAST, had not been published. This method was developed for easy and rapid screening of third-generation cephalosporin-resistant bacteria at our hospital and is not standardized. However, CLSI M100 ED32 published a test method for direct disk diffusion from positive blood cultures to determine susceptibility to ceftriaxone and ceftazidime 8–10 h after incubation in Enterobacterales [9]. CPDX rapid screening differs from the CLSI M100 procedure for testing disk diffusion directly from positive blood culture broth in that the incubation time is 6 h, one drop of blood culture broth is dispensed on Chocolate Agar EX II with vancomycin, streaked with an inoculating loop, and CPDX disks are used. Although this method was shown to be similar in accuracy to RAST, it is necessary to follow the CLSI M100 procedure for the rapid detection of resistant organisms using a standardized method. Although RAST requires MALDI-TOF MAS for the identification of bacterial species, the CLSI M100 procedure does not require identification of bacterial species. Therefore, by following the CLSI M100 procedure, the test results can be reported as a standardized method without the need to introduce equipment such as MALDI-TOF MS, which is expected to be introduced at many facilities.

The primary outcome of this study was that the proportion of cases wherein the administration of susceptible antimicrobial agents was modified within 24 h of blood culture -positive reports, significantly increased, despite 67.9% of cases reporting the results of CPDX rapid screening. Rapid phenotypic susceptibility testing has reduced time‐to‐appropriate antibiotic therapy [14, 15, 22], and this study showed similar results. Therefore, intervention with AST using CPDX rapid screening may contribute to appropriate antimicrobial use.

In *E. coli* and *K. pneumoniae*, most third-generation cephalosporin-resistant strains are ESBL-producing bacteria [1], and the first-line agents for ESBL-producing bacteria are carbapenems [23]. In the CPDX screening group, carbapenems were selected in most cases that were modified to a susceptible antimicrobial agent, suggesting that an appropriate antimicrobial agent was selected. However, several cases have been reported for cephamycins. Cephamycins are not recommended as a treatment for ESBL-producing bacterial infections [23]; however, several retrospective cohort studies comparing them to carbapenems have demonstrated their effectiveness [24–26]. Therefore, cephamycins are often used in Japan for urinary tract infections caused by ESBL-producing bacteria, and cephamycin has been selected for several cases. Thus, cephamycins and oxacephems, which are considered effective against bacteremia caused by ESBL-producing bacteria, are available in Japan and are administered as an alterative to carbapenems. In this study, 96.5% of the cases in which CPDX rapid screening was performed were cefmetazole susceptible. The use of an antimicrobial prescription protocol when third generation cephalosporin resistance is determined by the CPDX screening results used in this study may increase the use of carbapenems. However, in non-severe cases of uncomplicated urinary tract infections, in which the causative organisms are mostly *E. coli*, a protocol that suggests prescribing cephamycins or oxacephems may reduce the requirement for carbapenems prescriptions.

In the CPDX screening group, the method of identification of bacterial species was changed to MALDI-TOF MS during the study period. The introduction of MALDI-TOF MS increases the proportion of appropriate empirical treatments [27–30] and may affect the primary outcome. In these studies, the analysis included bacterial species other than *E. coli* and *K. pneumoniae*, and in *E. coli* and *K. pneumoniae* bacteremia, there were no cases [28] or a few cases [29, 30] of modification to carbapenems, the first-line agents in infections caused by ESBL-producing bacteria [23]. Therefore, MALDI-TOF-MAS is considered to have a small impact on the appropriate empirical treatment for ESBL-producing bacteremia, and its impact on the primary outcome in this study is considered to be small.

This study did not show improvement in secondary outcomes, such as 30-day mortality, hospital mortality, length of hospital stays, and cost of intravenous antibiotic therapy. Tumbarello M et al. reported that inappropriate empiric therapy increased mortality compared to appropriate empiric therapy in bacteremia caused by ESBL-producing bacteria (59.5% vs. 18.5%) [8]. In RCTs examining the clinical impact of rapid genotypic susceptibility testing and rapid phenotypic susceptibility testing, the highest mortality was 12.3%, and these reports did not show an improvement in mortality [14–17]. In terms of mortality improvement, this effect is expected only in the patient population with high mortality. Therefore, no improvement in mortality was observed in this study. In addition, these studies have not shown an improvement in the length of hospital stays [14–17]. The effect of the methods for determining antimicrobial resistance on the day of positive blood culture on improving the length of hospital stays is considered to be small. Moreover, the cost of intravenous antibiotic therapy could not be improved because the length of hospital stays did not improve.

This study had several limitations. First, this was a retrospective study, and although we had confirmed that there were no significant differences in the characteristics of the patients, we may not know all the factors affecting the outcomes. Second, the setting of each group was divided by time period; factors other than CPDX rapid screening may have affected the outcomes. The introduction of MALDI-TOF MS increases the proportion of appropriate empirical treatments [28–30], and the change to MALDI Biotyper for bacterial identification during the study period may have had any impact on the primary outcome.

This is the primary study providing insights into the utility and accuracy of CPDX rapid screening. Although this study showed that using CPDX rapid screening for the early detection of third-generation cephalosporin-resistant *E. coli* and *K. pneumoniae* could contribute to appropriate antimicrobial use, prospective studies using screening tests based on standard methods published by the CLSI are needed.

## Data Availability

The datasets used and/or analyzed during the current study are available from the corresponding author on reasonable request.

## Abbreviations

CPDX: Cefpodoxime
CTX: Cefotaxime
ESBL: Extended-spectrum β-lactamase
RAST: Rapid antimicrobial susceptibility testing
MALDI-TOF MS: Matrix-assisted laser desorption ionization time-of-flight mass spectrometry
RCT: Randomized controlled trial
AST: Antimicrobial stewardship team
CLSI: Clinical and laboratory standards institute
CCI: Charlson comorbidity index
PBS: Pitt bacteremia score
